# Epigenetic Repression of miR-218 Promotes Esophageal Carcinogenesis by Targeting *ROBO1*

**DOI:** 10.3390/ijms161126062

**Published:** 2015-11-20

**Authors:** Miao Yang, Ran Liu, Xiajun Li, Juan Liao, Yuepu Pu, Enchun Pan, Yi Wang, Lihong Yin

**Affiliations:** 1 Key Laboratory of Environmental Medicine Engineering, Ministry of Education, School of Public Health, Southeast University, Nanjing 210009, China; 230129498@seu.edu.cn (M.Y.); ranliu@seu.edu.cn (R.L.); 220122800@seu.edu.cn (X.L.); 230129495@seu.edu.cn (J.L.); yppu@seu.edu.cn (Y.P.); 2 Huaian Center for Disease Control and Prevention, Huaian 223001, China; hypec@163.com (E.P.); wangyihuaian@163.com (Y.W.)

**Keywords:** miR-218, CpG methylation, esophageal cancer, *ROBO1*

## Abstract

miR-218, consisting of miR-218-1 at 4p15.31 and miR-218-2 at 5q35.1, was significantly decreased in esophageal squamous cell carcinoma (ESCC) in our previous study. The aim of this study was to determine whether aberrant methylation is associated with miR-218 repression. Bisulfite sequencing analysis (BSP), methylation specific PCR (MSP), and 5-aza-2′-deoxycytidine treatment assay were applied to determine the methyaltion status of miR-218 in cells and clinical samples. *In vitro* assays were performed to explore the role of miR-218. Results showed that miR-218-1 was significantly CpG hypermethylated in tumor tissues (81%, 34/42) compared with paired non-tumor tissues (33%, 14/42) (*p* < 0.05). However, no statistical difference was found in miR-218-2. Accordingly, expression of miR-218 was negatively correlated with miR-218-1 methylation status (*p* < 0.05). After demethylation treatment by 5-aza-2′-deoxycytidine, there was a 2.53- and 2.40-fold increase of miR-218 expression in EC109 and EC9706, respectively. miR-218 suppressed cell proliferation and arrested cells at G1 phase by targeting 3′ untranslated region (3′UTR) of roundabout guidance receptor 1 (*ROBO1*). A negative correlation was found between miR-218 and *ROBO1* mRNA expression in clinical samples. In conclusion, our results support that aberrant CpG hypermethylation at least partly accounts for miR-218 silencing in ESCC, which impairs its tumor-suppressive function.

## 1. Introduction

Esophageal cancer ranks eighth in cancer incidence and sixth in cancer mortality worldwide [1]. Most patients are asymptomatic until at advanced stages, resulting in poor outcomes. Accumulated studies suggest that epigenetic alterations parallel the histologic changes in the progression of this disease [2]. DNA methylation, one of the extensively-studied epigenetic modifications, represses transcription of the promoter regions in tumor suppressor genes and, therefore, inactivates these genes’ expressions [3]. miRNAs, a class of non-coding small RNAs, exert a regulatory role through different downstream mRNAs by perfect or imperfect base-pairing [4]. Like protein coding genes, miRNAs could also be regulated by aberrant DNA methylation [5]. Several tumor-suppressive miRNAs have been reported to be silenced by CpG hypermethylation in esophageal squamous cell carcinoma (ESCC), including miR-375 [6], miR-34a [7], and miR-129-2 [8]. These studies indicate that DNA methylation is one of the crucial reasons for miRNAs’ dysregulation in cancers.

miR-218 captured our attention because of its potential tumor-suppressive role and the presence of dense CpG islands in the promoter regions shared with its host genes. In our previous study, miR-218 was identified to be down-regulated in 128 pairs of ESCC tumor tissues compared with paired non-tumor tissues by miRNA microarray and RT-PCR [9]. In fact, reduced expression of miR-218 was also reported in gastric cancer [10], nasopharyngeal cancer [11], and colon cancer [12]. These studies indicate that miR-218 may function as a tumor suppressor gene. As an intronic miRNA, miR-218 is supposed to be excised from the same primary transcript and co-regulated with its host genes, *SLIT2* and *SLIT3* [13,14]. *SLIT2* and *SLIT3* are commonly found to be silenced by aberrant DNA hypermethylation in promoter regions in cancers [15,16]. Thus, miR-218 is supposed to be transcriptionally silenced by aberrant DNA methylation under the same regulatory mechanism. We speculate that the loss of miR-218 in ESCC is a result of CpG islands’ hypermethylation in promoter regions.

In this study, we assessed the methylation status of miR-218 CpG islands in cells and clinical samples using bisulphite sequencing, methylation specific PCR, and 5-aza-2′-deoxycytidine treatment assay, and determined that miR-218 were CpG hypermethylated in ESCC. Further, we demonstrated that miR-218 inhibited cell proliferation and arrested cell cycle at G1 phase by directly targeting 3ʹUTR of *ROBO1*. In summary, the results indicate that repression of miR-218 plays an essential role in ESCC tumorigenesis, which is at least partly due to CpG hypermethylation.

## 2. Results

### 2.1. miR-218 Repression in ESCC Is Associated with CpG Hypermethylation

**Figure 1:**
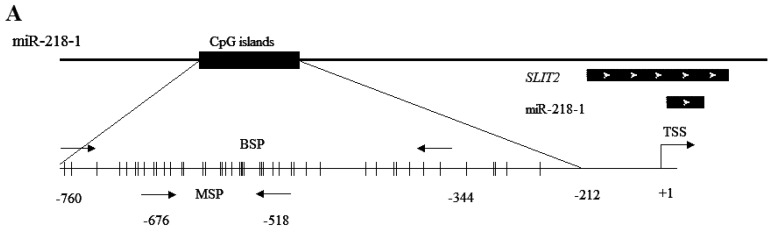

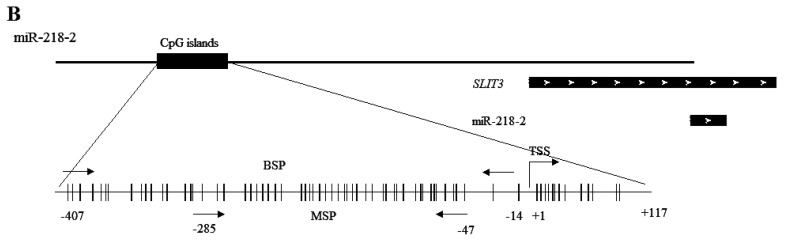
miR-218 is embedded within CpG islands. (**A**) Schematic illustration of the miR-218-1 gene embedded in the intron of *SLIT2* at 4p15.31, whose promoter region is embedded within a CpG island (black box). The transcription start site is designated as +1. The region −760 to −344 upstream of TSS was amplified for bisulfite sequencing, while −676 to −518 was amplified for methylation-specific PCR; and (**B**) schematic illustration of the miR-218-2 gene embedded in the intron of *SLIT3* at 5q35.1, whose promoter region is also embedded within a CpG island (black box). The region −407 to −14 upstream of TSS was amplified for bisulfite sequencing, while −285 to −47 was amplified for methylation specific PCR. Vertical bars represent CpG dinucleotides. Arrows indicate the direction of gene transcription. TSS: transcription start site; BSP: bisulfite sequencing analysis; MSP: methylation specific PCR.

**Figure 2:**
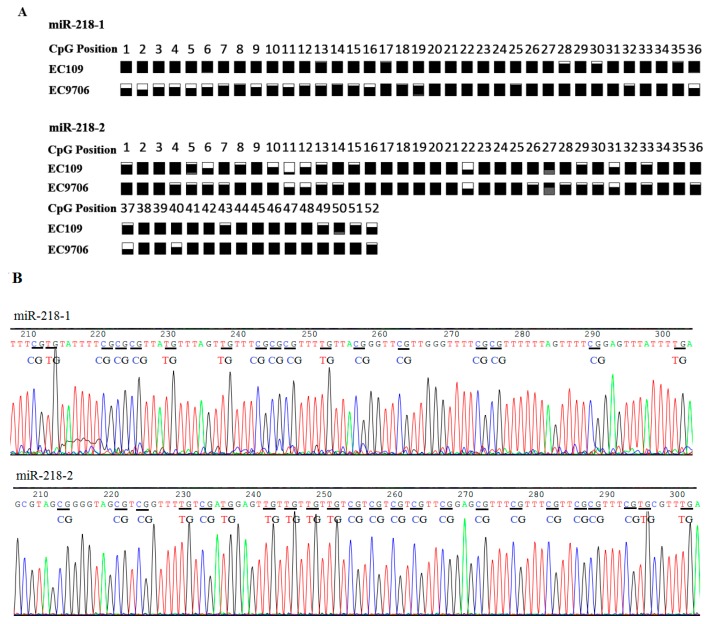
(**A**) miR-218 CpG methylation status in ESCC cell lines by bisulfite sequencing. White and black squares represent CpG site unmethylated and methylated, respectively. Grey squares represent CpG site not present. Partially squares filled with white and black represent semi-methylated CpG. Partially squares with grey represent that CpG site was not present in some clones; and (**B**) representative electropherogram from BSP analysis of miR-218 CGI methylation status. Lines in red, green, blue and black represent T, A, C, G, respectively. “CG” and “TG” pairs are indicated by “**—**“ in black. ESCC: esophageal squamous cell carcinoma.

**Figure 3:**
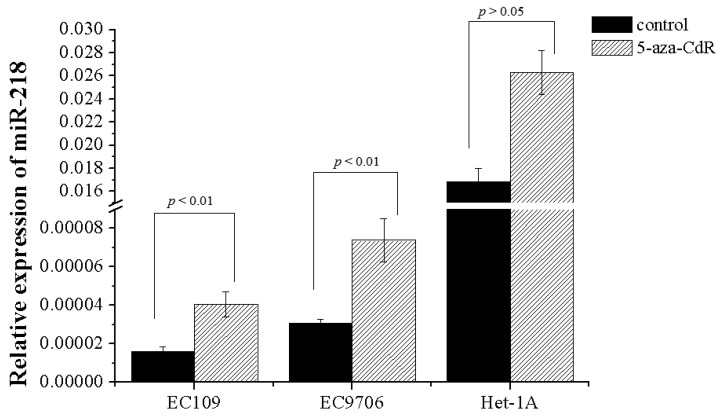
Effect of 5-aza-CdR treatment on miR-218 expression. ESCC cell lines were cultured in the absence or presence of 5-aza-CdR for 72 h. miR-218 expression level in demethylation treated ESCC cells including EC109 and EC9706 were significantly increased compared with those in control cells, while no difference in Het-1A.

CpG islands distribution in the promoter regions of miR-218 shared with host genes are detected by CpG Island Searcher Software (Available online: http://www.uscnorris.com/cpgislands2/cpg.aspx). Dense CpG islands are present at the position (−760 to −212) upstream to the transcription start site (TSS) in miR-218-1, and at the position (−407 to +117) to the TSS in miR-218-2 (Figure 1). Bisulfite sequencing analyses were applied to detect the methyaltion status of CpG sites of miR-218. Results showed that both of miR-218-1 and miR-218-2 were CpG-methylated in EC109 and EC9706 (Figure 2). To further elucidate whether miR-218 is epigenetically repressed, EC109 and EC9706 cells were treated with 5-aza-2′-deoxycytidine. The expression level of miR-218 in EC109 and EC9706 were recovered accordingly by 2.53- and 2.40-fold respectively after 5-aza-CdR treatment (Figure 3).

**Figure 4:**
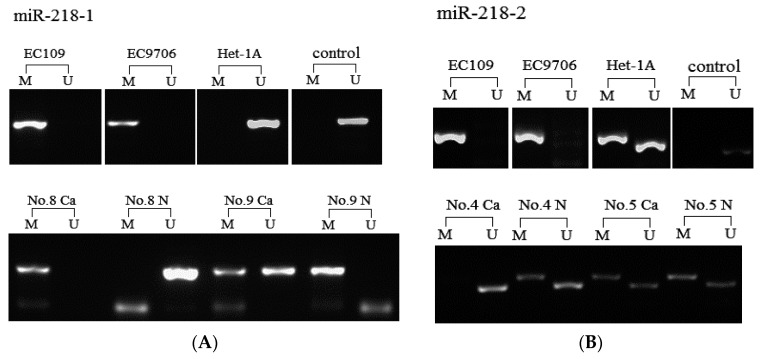
Detection of aberrant hypermethylation of the miR-218 by MSP analysis in cell lines and tissue samples. “M” represents PCR amplification using primers specific for methylated samples. “U” represents PCR amplification using primers specific for unmethylated samples. “Control” represents negative control from healthy human peripheral blood. “Ca” and “N” represent tumor tissues and paired non-tumor tissues, respectively. (**A**) CpG methylation of miR-218-1 in cells and tissues; and (**B**) CpG methylation of miR-218-2 in cells and tissues.

Having confirmed that miR-218 were epigenetically silenced in ESCC cell lines, we detected the methylation status of miR-218 in a total of 42 pairs tissues and cell lines EC9706, EC109, Het-1A using methylation specific PCR. miR-218-1 was found fully CpG-methylated in both EC9706 and EC109, while unmethylated in Het-1A (Figure 4A). CpG methylation of miR-218-1 frequently occurred in 34 cancer tissues, while only 14 paired non-tumor tissues were methylated (*p* < 0.05) (Figure 4A). For miR-218-2, it was also found fully methylated in two ESCC cell lines, while semi-methylated in Het-1A (Figure 4B). However, there is no difference in miR-218-2 methylation status between tumor tissues (69%, 29/42) and paired non-tumor tissues (60%, 25/42) (Figure 4B). The results strongly indicated the hypermethylation of miR-218 was associated with ESCC.

**Figure 5:**
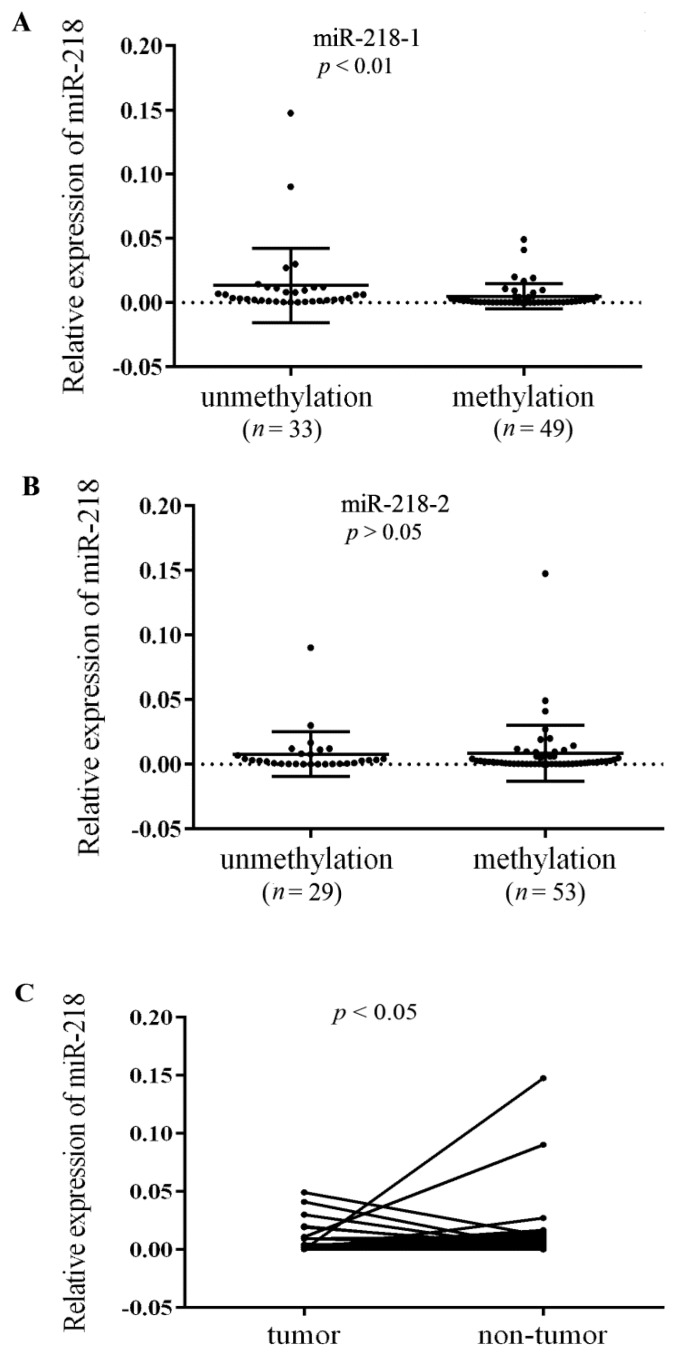
Aberrant DNA hypermethylation represses miR-218 expression. Expression of (**A**) miR-218-1; and (**B**) miR-218-2 were detected in methylated samples and unmethylated samples by qRT-PCR. Black lines represent error bars and mean values; (**C**) Expression of miR-218 in tumor tissues and paired non-tumor tissues.

Further, the correlation of miR-218 CpG methylation status and miR-218 expression was assessed in 41 ESCC tumor tissues and paired non-tumor tissues. In accordance with the reduced expression of miR-218 in cancer tissues, a significant downregulation of miR-218 in miR-218-1 methylation group (including full methylation and semi-methylation) than that in unmethylation group was detected (*p* < 0.01) (Figure 5A). While there is no such correlation in miR-218-2 (Figure 5B). Moreover, miR-218 was significantly decreased in tumor tissues compared with paired non-tumor tissues (*p* < 0.05) (Figure 5C). Collectively, the results suggest that hypermethylation of CpG islands especially miR-218-1 CpG hypermethylation is responsible for miR-218 silencing in ESCC.

### 2.2. miR-218 Suppresses Cell Proliferation by Arresting Cells at G1 Phase in ESCC

**Figure 6:**
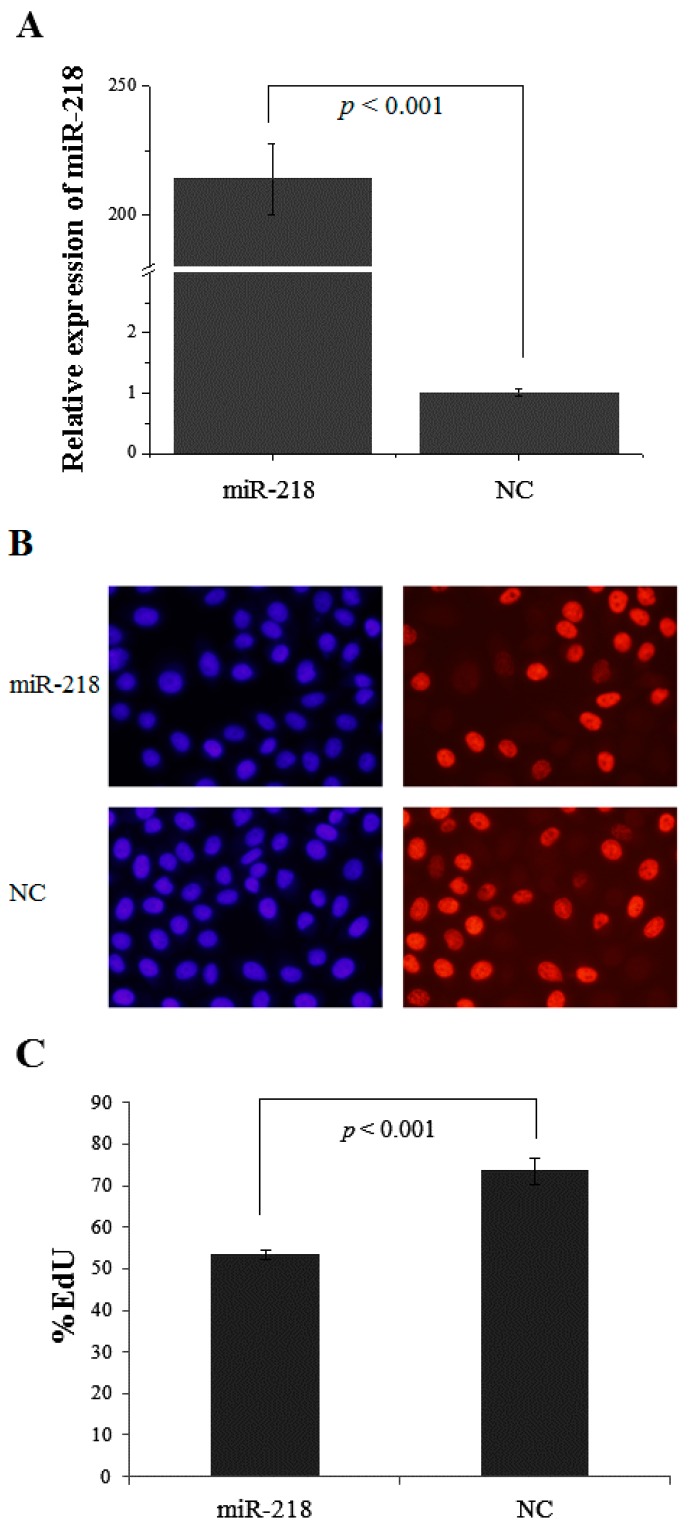
miR-218 suppresses cell proliferation. (**A**) MiR-218 expression in EC109 cells increased significantly when treated with 30 nM *miR-218* mimic compared with the negative control (NC); (**B**) Cells labeled in red after reaction of EdU (5-ethynyl-2′-deoxyuridine) and Apollo represent proliferative cells. Cell nuclei stained with Hoechst 33342 (blue) represent the total cells. The images are obtained by fluorescence microscope; (**C**) The percentage of proliferating cells was significantly increased (*p* < 0.001) in EC109 cells treated with miR-218 mimic than those in control cells.

**Figure 7:**
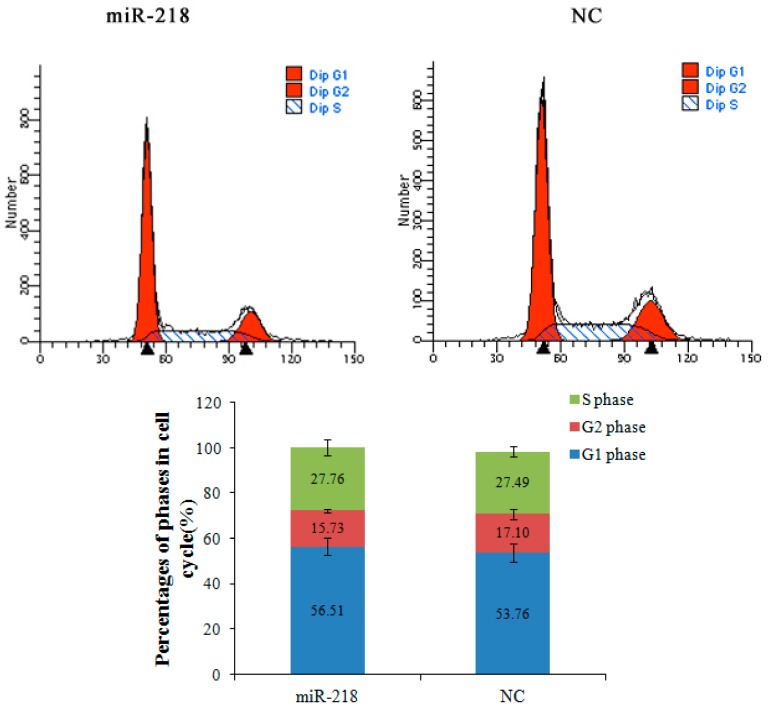
miR-218 arrested cell cycle at G1 phase. Flow cytometry analysis was performed to determine the constitution of cell cycle in miR-218 mimic treated cells and negative control cells. Histograms represent percentages of cells in each cell cycle phase. The cell percentage at different phases showed a cell cycle arrest in G1 phase when treated with miR-218 mimic (*p* < 0.01). NC: negative control.

To explore the biological function of miR-218 in ESCC, gain of function analysis was performed. Transfection efficiency was detected by qRT-PCR in cells treated with miR-218 mimic and negative control (*p* < 0.001) (Figure 6A). Cells treated with miR-218 mimic displayed decreased proliferative ability measured by EdU assay (*p* < 0.001) (Figure 6B,C). Cell cycle assay was performed to investigate the mechanisms of miR-218 on cell growth. Cells treated with miR-218 mimic was found significantly arrested at the G1 phase (*p* < 0.01) (Figure 7). It suggests that reduced miR-218 expression might impair cell proliferation by way of disturbing the percentages of cells at each phase.

### 2.3. Identification of miR-218 Target in ESCC Cells

**Figure 8:**
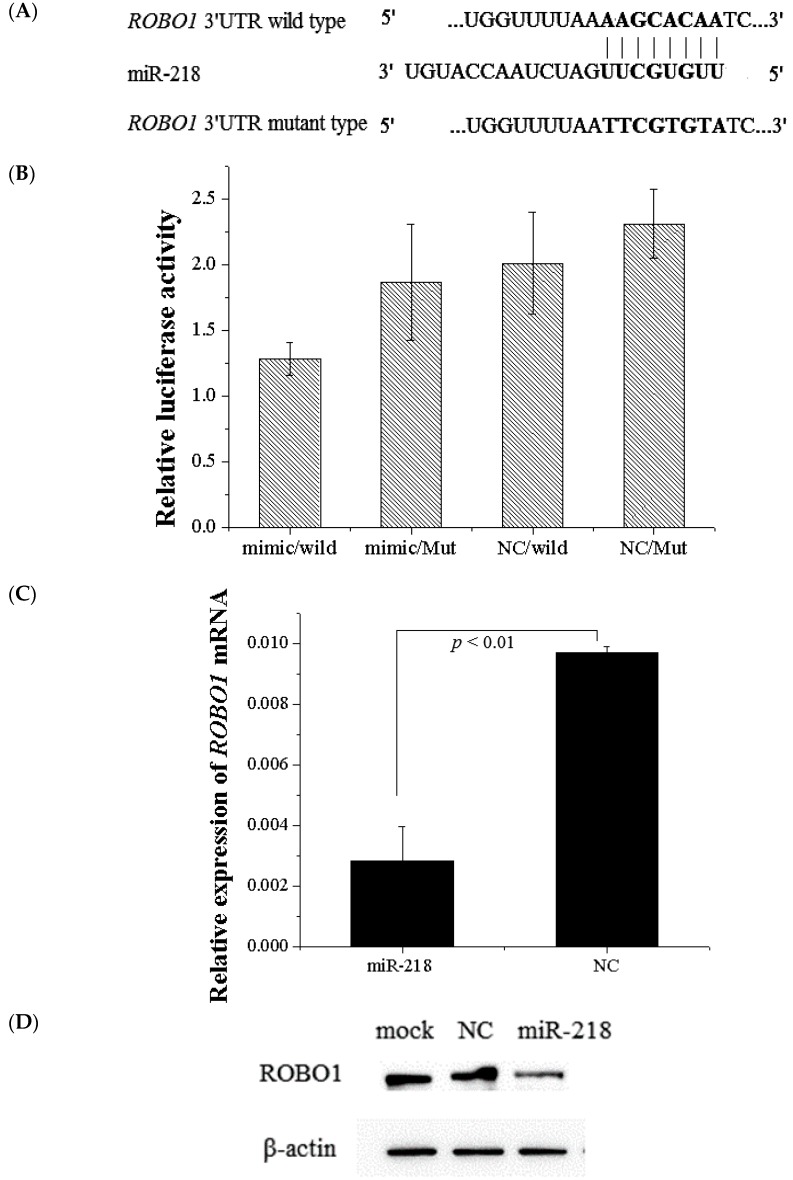
miR-218 targets 3′UTR of *ROBO1*. (**A**) The putative binding sites of miR-218 targeted the 3′UTR of *ROBO1*; (**B**) MiR-218 significantly down-regulated luciferase activity of wild type 3′UTR of *ROBO1* which suggests that miR-218 directly targets the 3′UTR of *ROBO1* (*p* < 0.001), but did not affect luciferase activity of mutant 3′UTR of *ROBO1*. WT: wild type 3′UTR of *ROBO1*; Mut: mutant 3′UTR of *ROBO1*; (**C**) Expression of *ROBO1* mRNA was detected by qPCR in EC109 treated with miR-218 mimic and negative control; (**D**) ROBO1 was detected by western blot in EC109 treated with miR-218 mimic and negative control. miR-218 significantly down-regulated *ROBO1* at both mRNA and protein levels. NC: negative control.

Potential miR-218 targets were predicted using target prediction programs including miRDB, TargetScan and Pictar. *ROBO1* was identified by all these prediction programs. To demonstrate that whether miR-218 directly regulates *ROBO1*, we employed a dual-luciferase reporter assay (Figure 8A). Results showed that miR-218 significantly inhibited nearly 45% expression of constructs of 3′UTR wild-type but not the mutant type, indicating that miR-218 directly regulates *ROBO1* by binding to 3′UTR of *ROBO1* (*p* < 0.001) (Figure 8B). Expression of *ROBO1* mRNA in cells treated with miR-218 mimic were decreased (fold change = 3.41) compared with negative control. Expression level of ROBO1 protein in miR-218 treated cells was accordingly decreased (fold change = 2.17) compared with negative control (Figure 8C,D).

**Figure 9:**
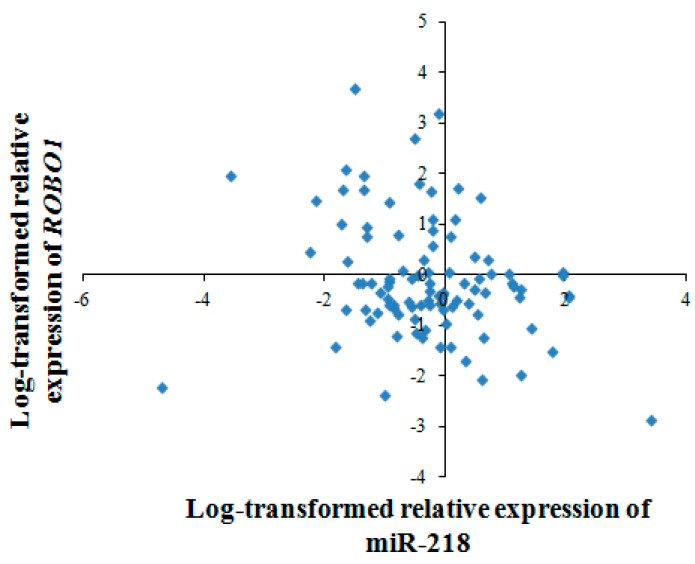
Association of miR-218 and *ROBO1* in ESCC tissues. The scatter plot showed a significant negative correlation between miR-218 and *ROBO1* (spearman coefficient = −0.258, *p* < 0.05). Expression levels of miR-218 and *ROBO1* are normalized by U6 and β-actin, respectively. Results are expressed as log-transformed relative gene expression (2^−ΔΔ*C*t^).

We further investigated the correlation between miR-218 and *ROBO1* in 97 pairs of ESCC tumor tissues and non-tumor tissues using quantitative RT-PCR. With log-transformed relative expression data (2^−ΔΔ*C*t^), it turned out that miR-218 expression was negatively correlated with *ROBO1* mRNA expression (spearman coefficient = −0.258, *p* < 0.05) (Figure 9). Taken together, *ROBO1* is a downstream gene of miR-218 in ESCC.

## 3. Discussion

DNA methylation in CpG islands is considered to be crucial in regulating gene activity and expression. Aberrant DNA methylation, including hypermethylation of tumor suppressor genes and hypomethylation of oncogenes occurs frequently in cancers [17], and growing evidences indicate that methylation status may serve as biomarkers that are more sensitive than genetic alterations [18]. miRNAs function as oncogenes or tumor suppressor genes by regulating distinct downstream target mRNAs. However, the mechanisms on miRNA dysregulation remain unclear. Katarzyna *et al.* [19] showed that miR-31 is regulated epigenetically in breast cancer together with its host gene lncRNA *LOC554202*. Cui *et al.* [7] found that miR-34a is epigenetically inactivated in esophageal cancer. These studies suggest that the aberrant CpG methylation in miRNA promoter regions may contribute to their dysregulation in tumors [20].

In our previous study, miR-218 was found significantly decreased in ESCC by microarray and RT-PCR [9]. miR-218 is located within the introns of *SLIT2* and *SLIT3*, whose promoter regions are frequently hypermethylated in cancers, including ovarian cancer [21], breast cancer [22,23], and cervical cancer [24]. The transcription of intronic miRNAs are deemed to coincide with their host genes, which suggests that they share the same regulatory sequences and can be regulated under the same mechanisms [25]. Intronic miR-335 is reported to be co-regulated with host gene *MEST* by promoter hypermethylation in hepatocellular carcinoma [26]. miR-342 was epigenetic silenced with host gene *EVL* in colorectal cancer [27]. miR-218 is found to be frequently down-regulated in different cancers [10,28], which is consistent with our previous findings. Thus, we speculated miR-218 may be silenced by aberrant CpG methylation together with host genes. In this study, bisulphite sequencing and methylation-specific PCR were performed to evaluate the methyaltion status of CpG islands in miR-218 promoter regions. BSP analysis showed that CpG islands of both miR-218-1 and miR-218-2 were hypermethylated in ESCC cells, while unmethylated and semi-methylated, respectively, in normal esophageal epithelial cell Het-1A. MSP analysis showed that miR-218-1 displayed higher CpG methylation rations in ESCC tumor tissues than that in paired non-tumor tissues, while no such statistical difference was found in miR-218-2. More importantly, we found that miR-218-1 CpG hypermethylation was correlated with miR-218 expression, and miR-218 can be restored in ESCC cells with treatment of DNA demethylating agent 5-aza-2′-deoxycytidine. Therefore, it indicates that miR-218 was epigenetically regulated in ESCC. Apart from DNA methylation, other mechanisms may underline miR-218 dysregulation in ESCC. Li [28] demonstrated histone acetylation may contribute to miR-218 down-regulation in breast cancer. Interestingly, human papillomavirus type 16 is reported to reduce the expression of miR-218 in cervical carcinoma [29]. Since human papillomavirus is closely involved in the development of ESCC [30], oncogenic HPV types may play a role in miR-218 dysregulation in ESCC. However whether these mechanisms contribute to miR-218 down-regulation in ESCC needs further investigation.

Previous studies indicate that down-regulation of miR-218 seems to be a frequent event in tumorigenesis [12,31]. miR-218 exerts its tumor-suppressive function through regulation of diverse downstream target genes in different cancers. Uesugi [32] demonstrated that loss of miR-218 activated mTOR-Akt signaling pathway through direct targeting *RICTOR* in oral cancer. In cervical cancer, miR-218 involves in focal adhesion pathway by directly targeting *LAMB3* [33]. In head and neck squamous cell carcinoma, lamin-332 is considered to be a direct downstream gene of miR-218 [34]. The role of miR-218 on cell cycle has been reported in several cancers. He [35] demonstrated miR-218 induces cell cycle arrest in the G2 phase of colon cancer cells. In breast cancer, miR-218 is found to prolong the G1 phase [28]. However, Tie [10] found miR-218 did not affect cell cycle in gastric cancer. It seems that the role of miR-218 on cell cycle varies in different cancers. And the specific roles of miR-218 in esophageal cancer have never been reported. In the present study, we found that restoring of miR-218 expression inhibited cell proliferation and arrested cell cycle at G1 phase. miR-218 is also reported to impact the migration and invasion abilities in cancers, which may be the reasons for miR-218 affecting malignant phenotypes [10,12,34]. Further, we identified *ROBO1* as a direct target gene of miR-218 through 3ʹUTR reporter assay. Expression of *ROBO1* were decreased when miR-218 was restored both at mRNA and protein levels. Correlation analysis further revealed the loss of miR-218 was negatively correlated with up-regulation of *ROBO1* in ESCC tissues and paired non-tumor tissues.

*ROBO1*, a member of the immunoglobulin gene superfamily and a receptor of *SLIT* family, is best known for the role of axon guidance in neuronal development [36,37,38]. Recent studies indicate that *ROBO1* is closely related to cancer progression. *ROBO1* is up-regulated in hepatocellular carcinoma [39] and colorectal cancer [40], which indicates its oncogenic role in these cancers. Additionally, *ROBO1* activates Wnt/β-catenin signaling pathway in intestinal tumor [41], thus, functions as a cancer-promoting oncogene. Whereas *ROBO1* could negatively regulate motility and invasiveness of primary prostate cancer cells and, thus, functions as a tumor suppressor to inhibit the progression of prostate cancer [42]. Similar tumor suppressor function was reported in intrahepatic cholangiocarcinoma [43]. It could be concluded that *ROBO1* appears to have conflicting roles in different cancers. In this study, we observed that *ROBO1* was significantly up-regulated in ESCC cells and clinical specimen. *ROBO1* mediates the downstream molecule *CDC42* through *srGAP*, while *CDC42* is involved in JNK signaling pathway. Thus, miR-218*/ROBO1* may be involved in *JNK* pathway through *CDC42*, which needs further investigation.

In summary, this study describes that the loss of miR-218 in ESCC is partly due to epigenetic hypermethylation at the promoter regions. The tumor suppressive effects of miR-218 on inhibiting cell proliferation and disturbing cell cycle were identified. *ROBO1* was a direct target gene of miR-218. The results support that the hypermethylation of CpG islands in miR-218 promoter regions was associated with ESCC tumorigenesis. Finally, there is great need to deeply explore the regulatory mechanisms and functions of key molecules.

## 4. Experimental Section

### 4.1. Clinical Samples

The total of 97 patients from the First People’s Hospital of Huaian were recruited between 2009 and 2010. All patients were diagnosed as primary squamous cell carcinoma of the esophagus. Tumor and paired non-tumor tissues (located ≥5 cm from the edge of tumor tissues) were collected and fresh frozen in an RNA Locker (Tiandz, Beijing, China) immediately after esophagectomy. Informed consent was obtained from every patient. The study was conducted according to protocols approved by the Southeast University Affiliated Zhongda Hospital Ethics Committee.

### 4.2. Cell Lines and Transfection

Human ESCC cell lines EC109, EC9706 and a human esophageal epithelial cell line Het-1A were used in this study. Cells were cultured in complete medium containing 100 U/mL each of penicillin and streptomycin (Sigma-Aldrich, Brooklyn, NY, USA) at 37 °C with 5% CO_2_.

For miR-218 mimic transfection, the miR-218 mimic (Ribobio, Guangzhou, China) was transfected into EC109 cells to transiently express miR-218. Cells (2 × 10^5^ per well) were seeded in a six-well plate with antibiotic-free medium the day before transfection, and transfected with 30 nM miR-218 mimic and 5 μL RNAiMAX (Life Technologies, Carlsbad, CA, USA) diluted by Opti-MEM medium (Life Technologies, Carlsbad, CA, USA). Transfection efficiency was determined by real-time RT-PCR after 48 h of incubation.

### 4.3. Total RNA Isolation and Quantitative Reverse Transcription PCR

Total RNA was extracted by using Trizol/chloroform according to protocols. After reverse transcription, real-time PCR was performed using StepOne Plus (Life Technologies, Carlsbad, CA, USA) and SYBR Green Master Mix (Toyobo, Osaka, Japan) according to the protocols. Relative expression of miR-218 was normalized against U6, while the expressions of *ROBO1* were normalized against β-actin. Primers sequences are described before [10].

### 4.4. DNA Isolation, Bisulphite Sequencing (BSP) and Methylation Specific PCR (MSP)

Genomic DNA was extracted using TIANamp Genomic DNA Kit (Tiangen, Beijing, China), and subjected to sodium bisulfite modification using EZ DNA Methylation Kit (Zymo, Irvine, CA, USA) as the manufacturer’s protocols. Unmethylated DNA obtained from healthy human peripheral blood was used as a negative control. CpG island searcher was used to screen for CpG islands which meet the following criteria: CG percentage > 55%; observed CpG/expected CpG > 0.65; length > 500 bp. For BSP, bisulfite-modified DNA was amplified by nested PCR. The PCR products were cloned into the pGEM-T Easy vector, and 10 clones were sequenced. For MSP, each sample were amplified with methylation-specific and unmethylation-specific primers. The products were examined by 3% agarose gel electrophoresis. The presence of a band in unmethylated samples without a methylation band was defined as unmethylated. However, when a band for methylated samples was present, and absent for an unmethylation band, we defined it as methylated (or semi-methylated if both unmethylated and methylated bands were present). Primer sequences are described before [23,44].

### 4.5. 5-Aza-2′-deoxycytidine Treatment

5-Aza-2′-deoxycytidine (Sigma-Aldrich, Brooklyn, NY, USA) was freshly prepared in PBS and filter-sterilized for use. Cells were treated with 10 μM 5-aza-2′-deoxycytidine for 72 h, and the medium was changed every three days. Total RNA were isolated for RT-PCR analysis.

### 4.6. Cell Proliferation Assay

Cell proliferation assay was performed using the EdU Apollo Imaging Kit (RiboBio, Guangzhou, China) as recommended in the manufacturer’s protocol. EdU (5-ethynyl-20-deoxyuridine) is a thymidine analogue that can be incorporated into DNA in proliferating cells [45]. After incubating with 50 μM EdU for 2 h, cells were fixed for 30 min and then washed with PBS, followed by 30 min incubation with EdU staining. After staining, cells washed and then labeled by Hoechst 33342 for another 30 min. Finally, cells were imaged and counted by a fluorescence microscope (FSX100, Olympus, Tokyo, Japan).

### 4.7. Cell Cycle Assay

After 48 h of transfection, cells were harvested and fixed at 4 °C overnight. The next day, cells were heated in 37 °C water bath with 100 μL RNase A. After 30 min of incubation, cells were stained with PI and analyzed by flow cytometry (Becton Dickinson, San Jose, CA, USA).

### 4.8. Western Blot

Total protein were extracted using RIPA buffer (Beyotime, Haimen, China) and protease inhibitors (Sunshinebio, Nanjing, China). 20 μg protein were separated by SDS-PAGE and transferred onto polyvinylidene difluoride (PVDF) membrane. Membranes were blocked by 5% non-fat milk for 1 h and incubated overnight at 4 °C with primary antibodies. The next day, membranes were washed with TBST buffer and incubated with secondary antibodies conjugated to horseradish peroxidase. Immunoreactive bands were visualized and analyzed using a chemiluminescent substrate (Thermo Fisher Scientific, Grand Island, NY, USA) and automatic chemical luminescence/fluorescence image analysis system (Tanon, Shanghai, China). The antibodies used in this study were mouse monoclonal β*-*actin (1:800, Boster, Wuhan, China), rabbit polyclonal *ROBO1* (1:200, Abcam, Cambridge, MA, USA), anti-mouse antibodies for monoclonal primary antibodies and anti-rabbit antibodies for polyclonal primary antibodies (1:5000, Santa Cruze, Dallas, TX, USA).

### 4.9. 3′UTR Luciferase Reporter Assay

Human *ROBO1* 3′UTR were inserted into pmiR-RB miRNA reporter vector (Ribobio, Guangzhou, China). Seed-matching sequences in *ROBO1* 3′UTR with miR-218 (AAGCACA) were replaced by TTCGTGT as a mutant control. For luciferase assays, cells were seeded in a 24-well plate with 5 × 10^4^ cells per well. After 24 h of incubation, cell were transfected with 100 ng plasmids and 30 nM miR-218 mimic or negative control. After 48 h, cells were analyzed using Dual-Glo luciferase reporter assay (Promega, Madison, WI, USA) and Mithras LB 940 (Berthold Technologies, Bad Wildbad, Germany). Relative renilla luciferase activity was normalized to luciferase activity.

### 4.10. Statistical Analysis

Data are expressed as means ± SD from three independent experiments. Differences in two groups were analyzed using the Student’s *t*-test, Mann–Whitney test, McNemar test. Correlation between genes was analyzed using Spearman correlation analysis. *p* < 0.05 was considered statistically significant.
